# Integrative Taxonomy Reveals a New Species of *Opsariichthys* (Teleostei, Cypriniformes) from the Pearl River Basin in Guangxi Zhuang Autonomous Region, China, with Synonymization of *O. minutus* †

**DOI:** 10.3390/ani16203193

**Published:** 2026-10-11

**Authors:** Yan-Wei Huang, Feng Lin, Jia-Jun Zhou, Ming-You Li, Lang Gui, Jin-Quan Yang

**Affiliations:** 1 Shanghai Universities Key Laboratory of Marine Animal Taxonomy and Evolution, Shanghai Ocean University, Shanghai 201306, China; 13823379148@163.com (Y.-W.H.); myli@shou.edu.cn (M.-Y.L.); lgui@shou.edu.cn (L.G.); 2 Guangxi Key Laboratory of Aquatic Genetic Breeding and Healthy Aquaculture, Guangxi Academy of Fishery Sciences, Nanning 530021, China; 3 Engineering Research Center of Hongshui River Rare Fish Conservation, Nanning 530021, China; 4 Zhejiang Forest Resource Monitoring Center, Hangzhou 310020, China; cnwaters@foxmail.com

**Keywords:** cytochrome *b*, D-loop, molecular phylogenetics, morphology, taxonomic revision, xenocyprididae

## Abstract

The taxonomy of *Opsariichthys* Bleeker, 1863 in southern China is complicated by conflicting morphological and molecular evidence, particularly the status of *O. minutus* Nichols, 1926, long treated as a junior synonym of *O. bidens* Günther, 1873 until its revalidation. To resolve this, we adopted an integrative approach combining D-loop sequencing to anchor specimens to the *O. minutus* lineage (supplementing scarce GenBank data with vouchers bearing undulating jaw margins), genus-wide cytochrome *b* (cyt *b*) phylogenetics to avoid circular reasoning, and detailed comparative morphology. D-loop analyses revealed that all specimens with undulating jaw margins formed two clades: Fujian Province populations clustered with *O. bidens*, while Guangxi Zhuang Autonomous Region populations formed a distinct monophyletic lineage. Cyt *b* phylogenetics confirmed Fujian populations shared <2.0% uncorrected p-distances with *O. bidens* and showed extensive morphological overlap, reaffirming *O. minutus* as a junior synonym of *O. bidens*. In contrast, Guangxi populations from the Xijiang River basin (middle and upper Pearl River system, including the Hongshuihe, Guijiang and Liujiang Rivers) formed a distinct monophyletic lineage with 7.4–15.9% uncorrected p-distances from congeners, diagnosed by 49–55 lateral-line scales, two pharyngeal tooth rows, and prominent lower-jaw tubercles arranged in 1–2 rows in breeding males. This lineage is described as a new species of *Opsariichthys*, with its type locality in the Hongshuihe River. Collectively, our findings not only resolve a long-standing taxonomic puzzle but also underscore the efficacy of integrative taxonomy in uncovering cryptic diversity within the highly biodiverse yet understudied Pearl River basin.

## Simple Summary

Small, colorful freshwater fish of the genus *Opsariichthys* inhabit rivers across China, Japan, the Korean Peninsula, and Vietnam. For decades, taxonomists have debated whether populations previously referred to as *O. minutus* from Fujian and Guangxi represent a single widespread species or multiple unrecognized lineages, due to their nearly indistinguishable jaw morphology. In this study, we integrated DNA sequencing and comparative morphology to resolve this taxonomic puzzle. We found that Fujian populations are conspecific with the widespread *O. bidens*, while Guangxi populations (from the Xijiang River basin, including the Hongshuihe, Guijiang, and Liujiang Rivers) represent a new species named *Opsariichthys baguiensis* sp. nov., with its type locality in the Hongshuihe River. Unlike closely related species with wavy-edged jaws, the new species is diagnosed by a higher lateral line scale count and prominent nuptial tubercles on the lower jaw (notable in breeding males). Our results clarify fish diversity in southern China and highlight the value of integrative taxonomy for biodiversity research in the Pearl River basin.

## 1. Introduction

The East Asian genus *Opsariichthys* Bleeker, 1863 (Xenocyprididae, Cypriniformes) is primarily distributed in upland streams across the majority of Chinese river systems, with no records from the arid northwestern and high-plateau regions [1]. Species delimitation within the genus has historically been based on the undulate-interdigitate morphology of the upper jaw margin. Following the synonymization of several earlier-described taxa, *O. bidens* Günther, 1873 (originally described from Shanghai) [2] has long been treated as the sole widespread continental species [3,4], a taxonomic framework retained in subsequent major revisions [1,5].

Recent molecular phylogenetic and integrative taxonomic studies, however, have challenged this long-standing arrangement. Notably, contemporary work has demonstrated that traditional diagnostic characters, such as the undulate-interdigitate upper jaw margin, exhibit marked phenotypic plasticity, whereas stable, previously underutilized traits—particularly distinct, independent transverse dark lateral stripes, a diagnostic character now adopted to differentiate *Opsariichthys* from the genus *Zacco* Jordan & Evermann, 1902—and mitochondrial markers have proven far more reliable for species delimitation [6,7]. Consequently, several former synonyms have been reinstated and numerous new species described, underscoring the critical role of integrative approaches in resolving diversity within the genus [8,9,10,11,12].

Within continental East Asia south of the Yangtze River, the undulate-interdigitate jaw-margin group was also long treated as a single species, *O. bidens*—a view overturned only by the integrative revisions noted above. Huynh and Chen [8] reinstated *O. minutus*, distributed in Fujian Province, and Chen and Huang (2008) [13] reinstated *O. hainanensis* Nichols & Pope, 1927 [14] from Hainan Island, both on the basis of integrative evidence. In addition, two species from northern Vietnam—*O. dienbienensis* and *O. songmaensis*—were described by Nguyen and Nguyen (2000) [15] on the basis of morphology. Among these, the taxonomic limits of *O. minutus* Nichols, 1926 [16] are particularly contentious and are examined in detail below.

Originally described from two small, incomplete specimens collected in Fujian Province, the taxon was subsequently relegated to a junior synonym of *O. bidens*, primarily because its curved (undulate-interdigitate) jaw margin was interpreted as consistent with the diagnostic condition of the latter [1,3,4,17]. Huynh and Chen [8], while describing *O. duchuunguyeni* Huynh & Chen, 2013 from northern Vietnam, directly treated *O. minutus* as valid based on D-loop sequence divergence and comparative morphology against *O. bidens* and other congeneric species as well as their new taxon. Subsequent studies extended this taxonomic assignment to populations from the Pearl River basin in Guangxi, with the associated mitochondrial cytochrome *b* (cyt *b*) sequences subsequently released [9,18]. Although molecular evidence has suggested that *Opsariichthys minutus* may represent a valid species [9,18], the phylogenetic relationships between the type-locality population in Fujian and populations from other regions of southern China (particularly the Xijiang River basin) have not yet been systematically assessed, and the actual distribution range as well as the intraspecific genetic diversity of this species remain poorly understood. Recent studies have revealed considerable cryptic diversity within the genus *Opsariichthys* [10,11], highlighting the necessity of re-evaluating the different geographic populations that have long been referred to *O. minutus*.

To address this taxonomic uncertainty, the aim of the present study was to re-evaluate the taxonomic status of *Opsariichthys* populations from Fujian and Guangxi through an integrative taxonomic approach combining molecular phylogenetics with detailed comparative morphology. Specifically, we tested (i) whether populations referred to *O. minutus* in Fujian are distinguishable from *O. bidens*, and (ii) whether the Xijiang River (Pearl River basin) population in Guangxi constitutes a distinct species. On the basis of this integrated assessment, we describe the Guangxi population as *Opsariichthys baguiensis* sp. nov. and treat the Fujian populations previously referred to *O. minutus* as a junior synonym of *O. bidens*. The rationale for the synonymization, the geographic and hydrological disjunction between the new species and the type locality of *O. minutus* (Fujian Province; precise locality not specified in Nichols, 1926 [16]), and the congruence of the morphological and molecular evidence are interpreted in the Discussion.

## 2. Materials and Methods

### 2.1. Specimen Sampling and Preservation

**Figure 1:**
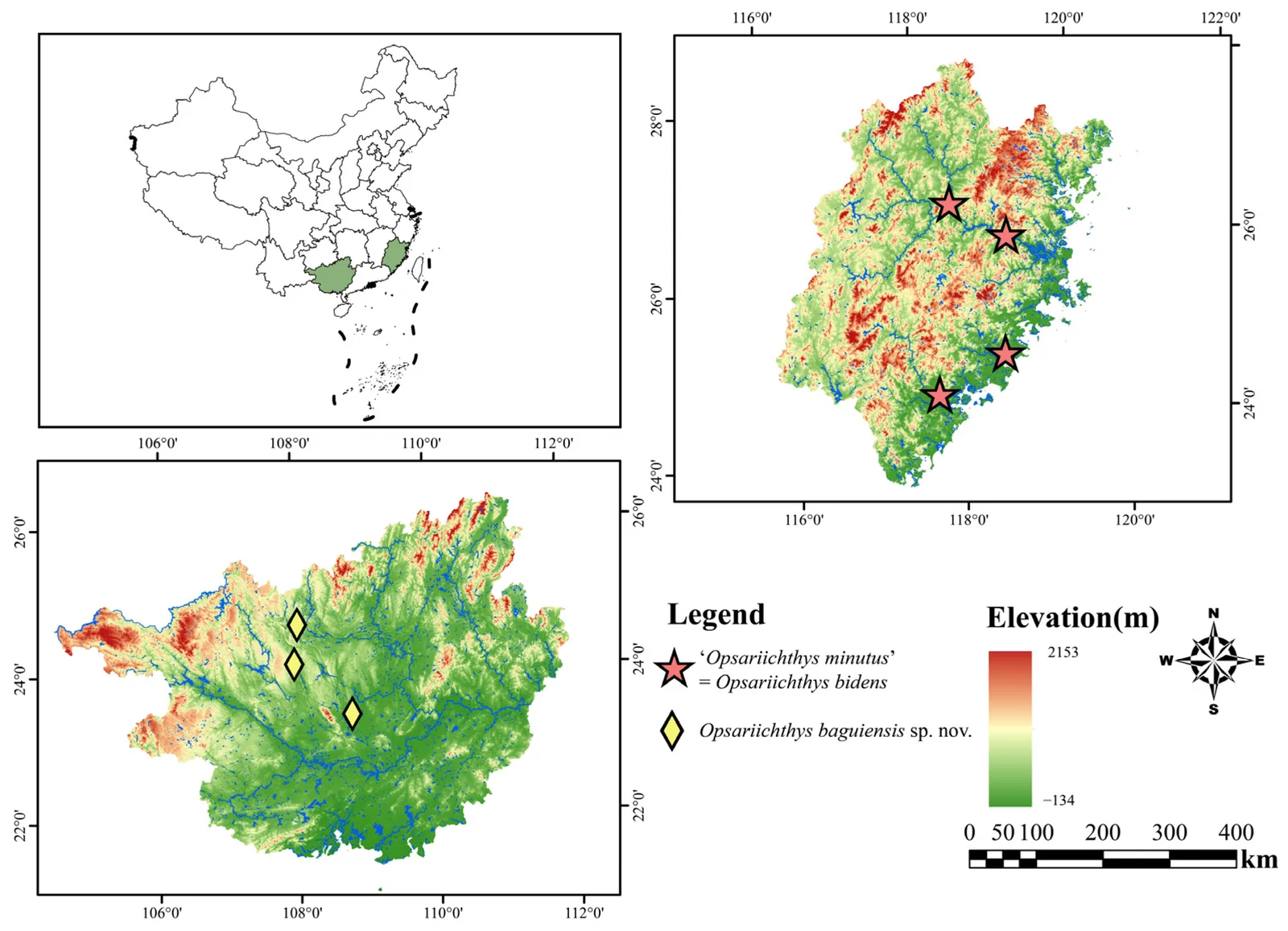
Sampling localities of the two *Opsariichthys* lineages included in the phylogenetic analysis: *O. bidens* (formerly identified as *O. minutus*) from Fujian Province, and *O. baguiensis* sp. nov. from Guangxi Zhuang Autonomous Region, China.

Specimens were collected from river systems in Guangxi and Fujian Provinces, southern China, between June 2024 and May 2026. Sampling localities encompassed the Xijiang River basin (Pearl River system) in Guangxi and the Minjiang, Jinjiang, and Jiulongjiang River basins in Fujian (Figure 1; full details including geographic coordinates in Table S1): Fujian Province—(1) Minjiang River: Fuzhou, 26.2817° N, 118.9075° E; (2) Minjiang River: Nanping, 26.7236° N, 118.2479° E; (3) Jinjiang River: Quanzhou, 24.9572° N, 118.6948° E; (4) Jiulongjiang River: Zhangzhou, 24.5888° N, 117.8179° E; Guangxi Zhuang Autonomous Region, Xijiang River basin (Hongshuihe River)—(5) Hechi, 24.7034° N, 108.0419° E; (6) Hechi, 24.1680° N, 107.9836° E; (7) Nanning, 23.4706° N, 108.8111° E. All fish specimens were captured using either dip nets or angling methods.

For molecular analyses, a small portion of muscle tissue was excised from the area below the dorsal fin on the right side of each specimen and preserved in 95% ethanol. Voucher specimens were fixed in 10% neutral-buffered formalin and later transferred to 60% ethanol for long-term storage. A total of 4 specimens are deposited in the Guangxi Zhuang Autonomous Region Academy of Fishery Sciences, with specimen numbers GXSC20260145–GXSC20260146 and GXSC20260296–GXSC20260297. The remaining specimens are deposited in the Fish Museum of Shanghai Ocean University (SHOU), Shanghai, China. Detailed information on paratypes will be provided in the species description section below.

### 2.2. DNA Extraction, PCR Amplification and Molecular Analysis

To anchor our specimens to the genetic lineage referred to as *Opsariichthys minutus* by Huynh and Chen [8], we first sequenced the mitochondrial D-loop region. As D-loop data in GenBank are scarce, we supplemented our dataset with publicly available sequences from vouchers exhibiting undulating jaw margins. Subsequently, we sequenced the cytochrome *b* (cyt *b*) gene and constructed a representative phylogenetic dataset by integrating our sequences with carefully selected GenBank accessions covering all recognized *Opsariichthys* species and major clades. To enable direct comparison with *O. bidens*, this cyt *b* dataset incorporated GenBank accessions of *O. bidens* from Jiangxi, Anhui, Zhejiang, Fujian, Guangdong, and Guangxi, covering the species’ geographic range in eastern and southern China; this coverage included populations from regions adjacent to the type locality (Shanghai)—specifically Jiangxi (Yangtze River system), Anhui (Qiantang River system), and Zhejiang (Qiantang River system)—representing the nearest available proximal populations, as *O. bidens* is no longer extant at the type locality itself. This sequential approach avoided circular reasoning by verifying the identity of *O. minutus* prior to conducting genus-wide comparisons using the more comprehensive cyt *b* dataset.

Genomic DNA was extracted from ethanol-preserved muscle tissue using a commercial Animal Genomic DNA Extraction Kit (Sangon Biotech, Shanghai, China). PCR amplifications were performed in 25 μL volumes containing 9.5 μL ddH_2_O, 1 μL of each primer (10 μM), 1 μL template DNA, and 12.5 μL 2× Taq PCR Master Mix. Thermal cycling consisted of an initial denaturation at 95 °C for 3 min; 35 cycles of denaturation at 94 °C for 30 s, annealing at 52 °C for D-loop or 54 °C for cyt *b* for 40 s, and extension at 72 °C for 1 min; followed by a final extension at 72 °C for 5 min. D-loop amplification used primers P-THRA (5′-AAA GCA TCG GTC TTG TAA TCC GAA G-3′) and CB-Phe-SR1 (5′-CAT CTT CAG TGC TAT GCT TT-3′) [8]; cyt *b* amplification used primers L14724 (5′-GAC TTG AAA AAC CAC CGT TG-3′) and H15915 (5′-CTC CGA TCT CCG GAT TAC AAG AC-3′) [19]. PCR products were purified and bidirectionally sequenced by Sangon Biotech. Raw chromatograms were assembled and manually edited using the SeqMan module of Lasergene (DNASTAR, v.7.1.0).

Sequence alignments were performed in MAFFT (v.7.5.2) [20] using the Auto strategy. In MEGA (v.11.0) [21], sequence variation analyses were conducted and pairwise and mean interspecific uncorrected p-distances were calculated to provide a quantitative basis for species delimitation. Molecular distinctness validation was conducted by comparing uncorrected p-distances of the Guangxi population against those of *O. bidens* (including the Fujian populations) and other recognized congeners against established cyprinid species-delimitation thresholds, together with the strongly supported monophyly recovered in the phylogenetic analyses. Molecularly diagnostic characters were identified by screening the cyt *b* alignment for fixed nucleotide differences between the Guangxi population and *O. bidens* (including the Fujian populations), and between the Fujian populations and *O. bidens* reference sequences, using MEGA; the same screening was repeated on the D-loop alignment for the Fujian populations versus *O. bidens*. The best-fit nucleotide substitution models were selected under the Bayesian Information Criterion (BIC) using ModelFinder (implemented in IQ-TREE v.2.0) [22]. For D-loop analyses, the HKY+F+G4 model was applied. For cyt *b* analyses, the TN+F+I+G4 model was used for Maximum Likelihood (ML) inference, while the GTR+F+I+G4 model was employed for Bayesian Inference (BI). Phylogenetic trees were then inferred using both ML and BI approaches. ML analyses were conducted in IQ-TREE (v.2.0) [23] with nodal support assessed using 1000 ultrafast bootstrap replicates. BI was performed in MrBayes (v.3.2.6) [24], running two independent Markov chain Monte Carlo (MCMC) chains for 10,000,000 generations and sampling every 1000 generations. The first 25% of sampled trees were discarded as burn-in, and convergence was considered achieved when the average standard deviation of split frequencies fell below 0.01. Resulting phylogenetic trees were visualized in FigTree (v.1.4.4) [25].

### 2.3. Morphological Analyses

Morphological comparisons were conducted primarily with *O. hainanensis* Nichols & Pope, 1927, as the focal group exhibits the key diagnostic trait of undulating jaw margins characteristic of this species, coupled with largely adjacent geographic distributions. Comparative data for other congeners possessing this feature—including *O. bidens*, the Vietnamese species *O. dienbienensis*, and *O. songmaensis*—were obtained from the literature. Morphometric measurements were taken on the left side of each specimen using digital calipers (precision: 0.1 mm), with standard length (SL) as the baseline measurement. Methods for morphological and meristic character evaluation followed Peng et al. [10]. Meristic traits were abbreviated as follows: D, dorsal-fin rays; A, anal-fin rays; P1, pectoral-fin rays; P2, pelvic-fin rays; LL, lateral-line scales; LLa, scales above lateral line; LLb, scales below lateral line; PreD, predorsal scales; CPS, circumpeduncular scales.

Two-way hierarchical clustering and principal component analysis (PCA) were used to analyze morphological variation among *Opsariichthys* species. Clustering was based on mean values of 18 traits from six species: *O. baguiensis* sp. nov., *O. hainanensis*, and *O. minutus* measured in this study, plus *O. bidens*, *O. songmaensis*, and *O. dienbienensis* from the literature [8,10]. Because the literature data for *O. dienbienensis* and *O. songmaensis* [8] contained only 18 traits overlapping with our dataset, the clustering analysis used these 18 shared traits. Traits were Z-score standardized, and rows and columns were clustered using Euclidean distance and complete linkage. The heatmap shows red for above-mean and green for below-mean values. The morphological traits included in the heatmap are abbreviated as follows: BD (body depth), HL (head length), HD (head depth), HW (head width), SnL (snout length), ED (eye diameter), IW (interorbital width), CPL (caudal peduncle length), CPD (caudal peduncle depth), DL (dorsal-fin length), PtL (pectoral-fin length), PvL (pelvic-fin length), AL (anal-fin length), PDL (predorsal length), PPvL (prepelvic-fin length), PAL (preanal-fin length), DBL (dorsal-fin base length), and ABL (anal-fin base length). PCA used individual data from the three species measured in this study (*O. baguiensis* sp. nov., *O. hainanensis*, and ‘*O. minutus*’) plus *O. bidens* obtained from literature [10], totaling 21 morphological traits; *O. dienbienensis* and *O. songmaensis* were not included in PCA because only 18 shared traits were available. Each trait was Z-score standardized. Both two-way hierarchical clustering analysis [26] and PCA [27] were performed using R 4.5.1 [28].

## 3. Results

### 3.1. Molecular Phylogenetic Analyses

We analyzed D-loop and cyt *b* regions with *Aphyocypris chinensis* as the outgroup. The D-loop dataset (41 sequences: 23 new, 18 GenBank) gave a 930 bp alignment with 832 conserved and 96 variable sites (22 singleton, 74 parsimony-informative). The cyt *b* dataset (70 sequences: 25 new, 45 GenBank) gave a 1140 bp alignment with 656 conserved and 484 variable sites (95 singleton, 389 parsimony-informative). Screening of these alignments for fixed nucleotide differences identified 113 such positions between the Guangxi population (the new species) and *O. bidens* (including the Fujian populations and other drainages), providing a clear molecular diagnostic for species recognition; in contrast, zero fixed differences were observed between the Fujian ‘*O. minutus*’ populations and other *O. bidens* populations, and likewise zero based on D-loop sequences.

D-loop trees (ML and BI) showed congruent topologies with strong nodal support (ML bootstrap ≥ 90%, BI posterior probability ≥ 0.92; Figure S1). All specimens had undulating jaw margins and, within this D-loop dataset, fell into two clades. The *O. bidens* assemblage (corresponding to Clade II in the cyt *b* tree) comprised *O. uncirostris* sister to *O. amurensis* + *O. bidens* (including previously identified ‘*O. minutus*’ sequences); specimens from Fujian Province labeled as ‘*O. minutus*’ (Minjiang, Jinjiang, and Jiulongjiang River basins) grouped with reference sequences from Huynh and Chen [8] and GenBank *O. bidens* sequences from the Hanjiang and Yangtze River basins; the low genetic divergence between them suggests that *O. minutus* falls within the taxonomic scope of *O. bidens*. The Guangxi Xijiang lineage (corresponding to Clade III in the cyt *b* tree) consisted exclusively of specimens from the Pearl River system (Xi Jiang, Guangxi), all newly sequenced in this study, forming a strongly supported monophyletic group (ML/BI = 97/1); this clade is sister to a southwestern congeneric group (*O. hainanensis*, *O. dienbienensis*, *O. songmaensis*), together distinct from *O. bidens*.

**Figure 2:**
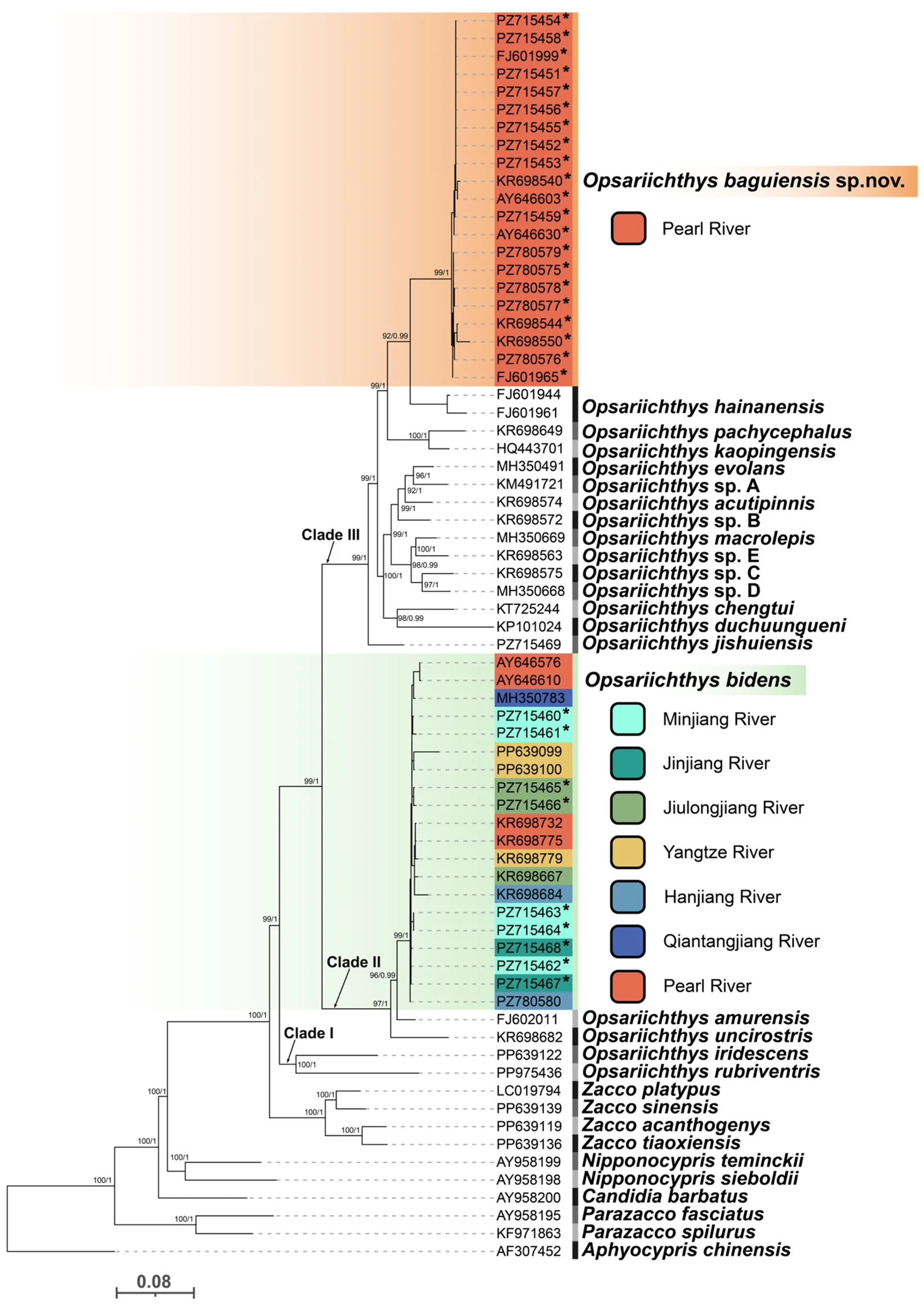
Phylogenetic relationships of Opsariichthyinae inferred from mitochondrial cytochrome *b* sequences. The ML tree is shown; BI analysis recovered congruent topologies. Nodal support values represent ML bootstrap proportions/BI posterior probabilities. The *O. bidens* assemblage and the new species (*O. baguiensis* sp. nov.) are distinguished by shading; drainage-specific colors are given in the legend at right. Asterisks (*) mark sequences previously identified as *O. minutus*, from both Fujian and Guangxi populations.

The cyt *b* dataset recovered highly congruent ML and BI topologies with strong nodal support (ML bootstrap ≥ 92%, BI posterior probability ≥ 0.99; Figure 2). All recognized *Opsariichthys* species and molecular lineages formed a strongly supported monophyletic group, resolving into three major clades (I–III). Clade I comprised *O. iridescens* and *O. rubriventris* as sister taxa, which together formed the sister group to Clades II–III. Clade II included the three species characterized by undulating jaw margins (*O. uncirostris*, *O. amurensis*, and *O. bidens*). Within this clade, all Fujian specimens previously identified as ‘*O. minutus*’ (Minjiang, Jinjiang, Jiulongjiang) clustered unequivocally with *O. bidens* sequences sourced from the Pearl River, Yangtze River, Qiantangjiang River, and adjacent river systems, confirming their phylogenetic placement within *O. bidens*. Clade III represented a distinct evolutionary lineage. All Guangxi specimens previously identified as ‘*O. minutus*’ (including both newly sequenced and GenBank sequences) formed a strongly supported monophyletic group—the new species—that was markedly divergent from *O. bidens*. Within Clade III, the Guangxi ‘*O. minutus*’ specimens (the new species) were sister to *O. hainanensis* (which also bears undulating jaw margins), while *O. jishuiensis* (with straight jaw margins) occupied a basal position; Clade III additionally comprised additional described species and previously identified molecular lineages, and was recovered as the sister group to Clade II, distinct from Clade I. Pairwise uncorrected p-distances revealed substantial divergence between the Pearl River lineage and other recognized congeners (7.4–15.9%), far exceeding the intraspecific variation observed within *O. bidens* (<2.0%) (Table S2) and approaching or surpassing levels typically considered indicative of species-level differentiation in cyprinid fishes, thus supporting its taxonomic recognition as a distinct species.

### 3.2. Morphological Comparisons

A two-way hierarchical clustering heatmap based on species means of 18 shared morphological traits across six species showed that *O. baguiensis* sp. nov. formed a cluster by itself and was clearly separated from the other five species (*O. bidens*, ‘*O. minutus*’, *O. hainanensis*, *O. songmaensis*, and *O. dienbienensis*) (Figure S2). The mean standardized value across traits for *O. baguiensis* sp. nov. was the lowest, and it appeared predominantly green in the heatmap, indicating that most of its morphological traits were below the overall average, forming a distinctive low-value combination. The remaining five species clustered into another cluster. Although ‘*O. minutus*’ was overall reddish and had the highest mean, it still clustered with species such as *O. bidens* and did not form an independent branch, morphologically supporting the treatment of ‘*O. minutus*’ as a junior synonym of *O. bidens*. Trait clustering divided the 18 traits into three clusters: cluster 1 (PtL, CPD, PvL, BD, HL, CPL, PPvL, PAL), cluster 2 (ED, DBL, ABL, DL), and cluster 3 (AL, IW, HD, HW, SnL, PDL). Traits within the same cluster showed similar patterns of high and low values across species, suggesting coordinated variation among these traits.

Principal component analysis (PCA) was conducted on four *Opsariichthys* species (*O. minutus*, *O. bidens*, *O. hainanensis*, and *O. baguiensis* sp. nov.) based on 21 morphological variables. The cumulative contribution of PC1 and PC2 was 70.62% (PC1 52.12%, PC2 18.50%), representing most of the information in the original data. In the principal component score plot (Figure S3), *O. hainanensis* was located at the positive extreme of PC1; *O. baguiensis* sp. nov. was mainly distributed in the lower-left quadrant (negative PC1 and negative PC2), clearly separated from the other groups; *O. minutus* was mainly distributed in the upper-left quadrant (negative PC1, positive PC2); while *O. bidens* was mainly distributed in the upper-left quadrant (negative PC1, positive PC2, with the Fujian populations extending toward the origin). *O. minutus* and *O. bidens* overlapped and were not distinguishable.

### 3.3. Taxonomic Account

**Figure 3:**
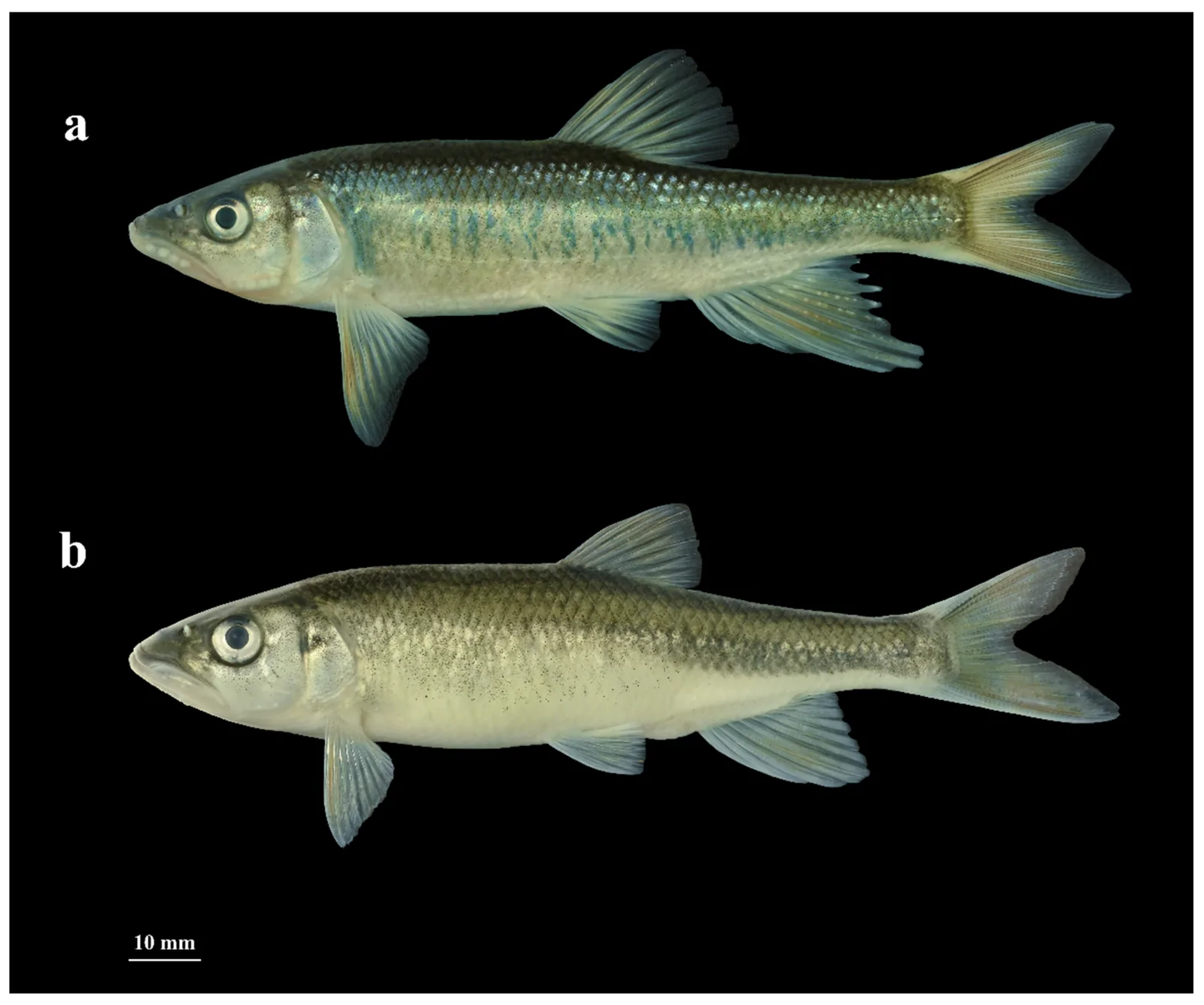
Photographs of preserved specimens of *Opsariichthys baguiensis* sp. nov. (**a**). Holotype, SHOU202605001, male, 107.4 mm SL; (**b**). Paratype, SHOU202603001, female, 104.3 mm SL.

**Figure 4:**
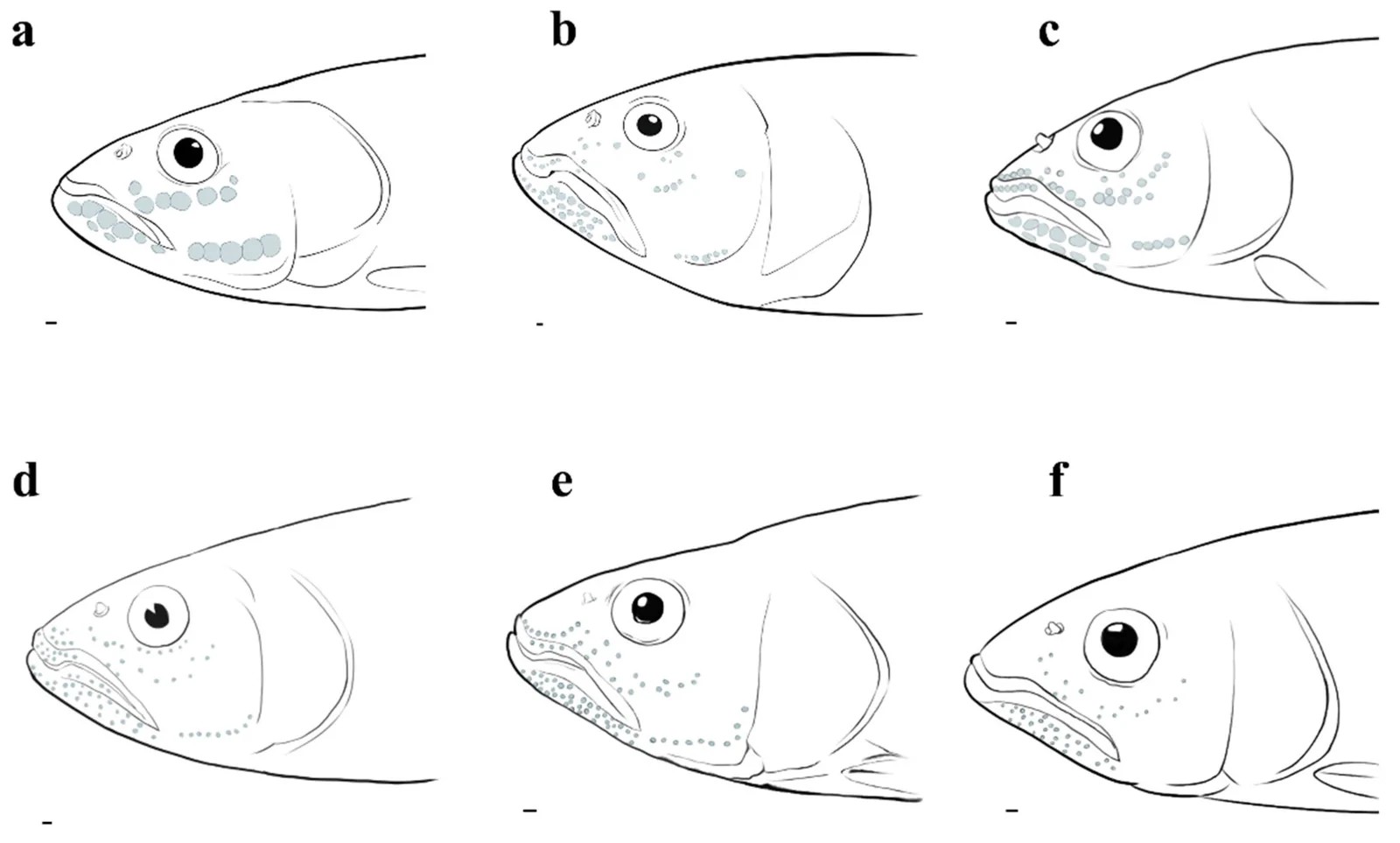
Comparisons of head tubercles among *Opsariichthys* species with undulating jaw margins. (**a**) *Opsariichthys baguiensis* sp. nov. (SHOU202504001, male, Hongshuihe River, Nanning City); (**b**) *Opsariichthys bidens* (SHOU202111001, male, Cao’ejiang River, Shengzhou City, Zhejiang Province); (**c**) *Opsariichthys hainanensis* (SHOU202602004, male, Changhuajiang River, Dongfang City, Hainan Province); (**d**) *Opsariichthys uncirostris*; (**e**) *Opsariichthys dienbieensis*; (**f**) *Opsariichthys songmaensis* (**d**–**f** modified from Huynh and Chen [8]). Scale bar = 1 mm.

**Figure 5:**
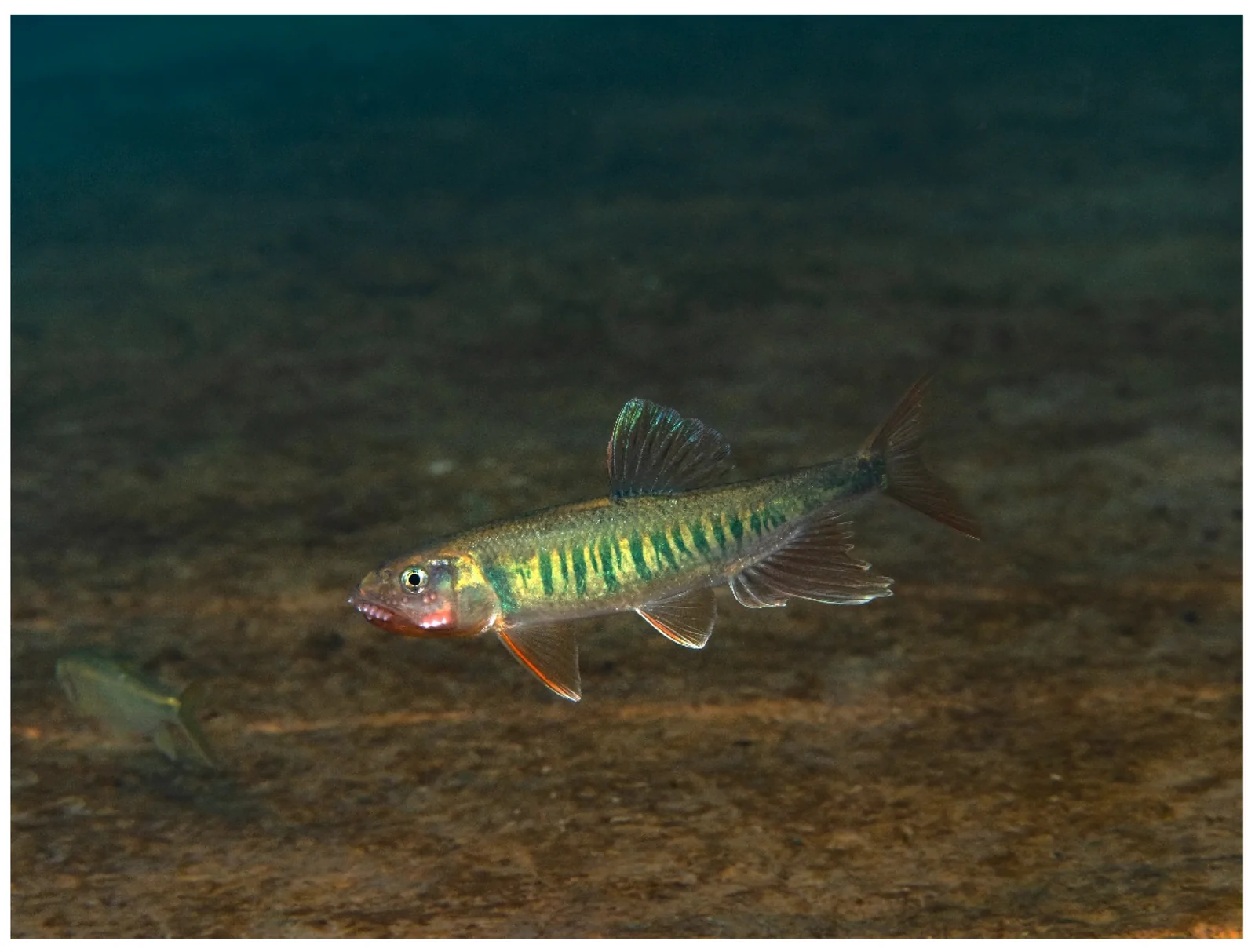
Habitat photograph of *Opsariichthys baguiensis* sp. nov. (male). The site is characterized by a relatively flat riverbed substrate.

**Figure 6:**
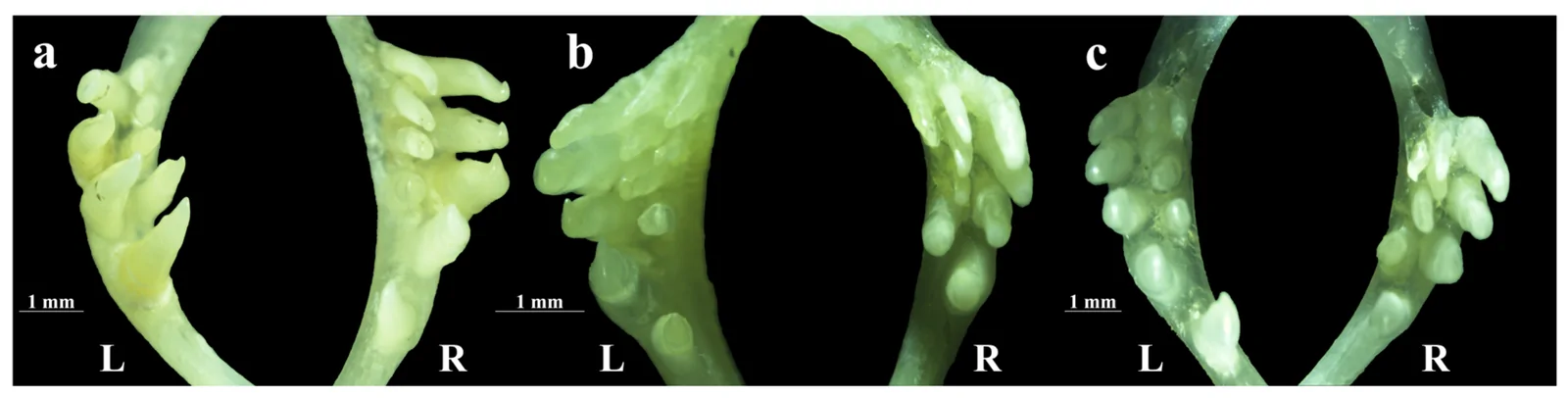
Pharyngeal tooth comparisons among *Opsariichthys* species with undulating jaw margins. (**a**) *Opsariichthys baguiensis* sp. nov., pharyngeal formula: 5·4/4·4; (**b**) *Opsariichthys bidens*, pharyngeal formula: 5·4·2/1·4·4; (**c**) *Opsariichthys hainanensis*, pharyngeal formula: 5·4·1/1·4·4. L, left pharyngeal teeth; R, right pharyngeal teeth.

***Opsariichthys baguiensis* sp. nov.**

urn:lsid:zoobank.org:act:A74E068B-FE71-4AB8-9F25 -EA663BA14980 (Figure 3, Figure 4a, Figure 5 and Figure 6a)

**Holotype.** SHOU202605001, male, adult, 107.4 mm SL (Figure 3), collected from Du’an Yao Autonomous County, Hechi City, Guangxi Zhuang Autonomous Region (Hongshuihe River, 24.1680° N, 107.9836° E) on 12 May 2026, by Jia-Jun Zhou.

**Paratypes.** SHOU202603001, female, adult, 104.3 mm SL, collected from Du’an Yao Autonomous County, Hechi City, Guangxi Zhuang Autonomous Region (Hongshuihe River, 24.0664° N, 108.0555° E) in March 2026, by Yue Cai; SHOU202605002, male, adult, 134.9 mm SL, collected from Jinxiu County, Laibin City, Guangxi Zhuang Autonomous Region (Liujiang River, 23.9425° N, 110.0752° E) in May 2026, by Feng Lin; GXSC20260145–GXSC20260146, male, adult, 91.3–95.1 mm SL, collected from Jinxiu County, Laibin City, Guangxi Zhuang Autonomous Region (Liujiang River, 24.1375° N, 110.0877° E) in May 2026, by Feng Lin; GXSC20260296–GXSC20260297, male, adult, 103.4–128.9 mm SL, collected from Jinxiu County, Laibin City, Guangxi Zhuang Autonomous Region (Guijiang River, 24.2515° N, 110.2677° E) in May 2026, by Feng Lin; SHOU202504001–SHOU202504017, male/female, adult, 85.6–120.6 mm SL, collected from Shanglin County, Nanning City, Guangxi Zhuang Autonomous Region (Hongshuihe River, 23.4706° N, 108.8111° E) in April 2025, by Jia-Jun Zhou; SHOU20241001–SHOU20241004, male/female, adult, 96.1–109.5 mm SL, collected from Jinchengjiang District, Hechi City, Guangxi Zhuang Autonomous Region (Liujiang River, 24.7034° N, 108.0419° E) in October 2024, by Jia-Jun Zhou.

**Diagnosis.** *Opsariichthys baguiensis* sp. nov. is readily distinguished from all congeners with straight jaw margins by possessing undulating jaw margins. Among congeners with undulating jaw margins, it differs by the following core unique combination of characters (summarized in Table 1): (1) lateral-line scales 49–55; (2) sexually mature males with 1–2 rows of prominent, densely packed, often basally fused tubercles on lower jaw (Figure 4); (3) cheek with 1–2 rows of large, even basally fused tubercles in mature males (Figure 4); (4) pharyngeal teeth arranged in two rows (Figure 6); (5) relatively elongate snout (36.5–40.1% HL, mean 38.1%); (6) comparatively short caudal peduncle (13.6–17.1% SL, mean 15.7%). Detailed morphological comparisons with other congeners with undulating jaw margins are provided in Table 2 and Table 3.

**Description.** Dorsal fin rays iii, 7; anal fin rays iii, 9–10; pectoral fin rays i, 13–14; pelvic fin rays i, 8–9; lateral-line scales 49–55; scales above lateral line 9 or 10; scales below lateral line 4; predorsal scales 17–21; circumpeduncular scales 15–20; pharyngeal teeth arranged in two rows, formula 5·4/4·4 (Figure 6a).

Body elongate, laterally compressed, abdomen rounded, without ventral keel. Body depth slightly less than head length. Snout blunt. Mouth oblique; upper jaw extending posteriorly to vertical through mid-orbit. Lower jaw slightly exceeding upper jaw, with distinct anterior protrusion articulating into median notch of upper jaw, forming undulating jaw margins. Maxillary barbels absent. Breeding tubercles well developed in sexually mature males: 1–2 rows of prominent, densely packed tubercles on lower jaw (Figure 4); cheek with 1–2 rows of large, even basally fused tubercles. Body covered with cycloid scales. Lateral line complete, descending obliquely below pectoral fin and continuing along the lower half of body to mid-lateral region of caudal peduncle. Dorsal-fin origin slightly anterior to or aligned with pelvic-fin origin. Pectoral fins slightly pointed distally, failing to reach pelvic-fin origin when adpressed. Pelvic fins failing to reach anus. Anus immediately anterior to anal-fin origin. Anal fin elongate: in mature males, longest rays extend beyond caudal-fin base; in females, they fail to reach it. Caudal fin forked, lobes pointed, upper and lower lobes subequal in length.

**Coloration.** Live individuals exhibit typical countershading, with the dorsum dark gray to black and the venter silvery white (Figure 5). Breeding males display well-developed nuptial coloration: lower jaw and cheek region orange-red; dorsal-fin rays pale orange with dark gray interradial membranes; pectoral and pelvic fins pale orange with outermost fin rays bright orange-red; anal and caudal fins pale orange with hyaline to lightly orange-tinged membranes. The flank bears 12–18 irregular bluish-green vertical bars, the first (posterior to operculum) longest, extending ventrally to the pectoral-fin base. Two bars on the caudal peduncle are distinctly broader than the others. Females lack distinct vertical bars, with all fins greenish-yellow.

In preserved specimens (10% formalin) (Figure 3), overall coloration fades to dark gray dorsally and grayish white ventrally; all fins grayish white, flank bars remaining visible but turning black.

**Distribution.** The new species is known from the Xijiang River Basin (middle and upper Pearl River system) in Guangxi Zhuang Autonomous Region, based on voucher specimens and GenBank cyt *b* sequences. Voucher specimens were collected from the Hongshuihe, Guijiang and Liujiang Rivers (Figure 1). GenBank cyt *b* sequences further confirm its occurrence in the Youjiang and Mingjiang Rivers.

**Habitat.** This species primarily inhabits streams with moderate flow velocities and relatively deep water columns. These habitats are characterized by clear water, substrates of small- to medium-sized pebbles and sand, and dense aquatic vegetation coverage.

**Etymology.** The specific epithet *baguiensis* is derived from “bagui” (八桂), the historical toponym of Guangxi Zhuang Autonomous Region, Latinized from Chinese pinyin with the locative suffix -*ensis* indicating origin from the type locality. We propose the Chinese vernacular name “八桂马口鱼 (Bā Guì Mǎ Kǒu Yú)” for this species.

## 4. Discussion

The taxonomy of *Opsariichthys* in southern China has been historically complicated by excessive reliance on phenotypically plastic traits, most notably the undulating jaw margin [1,6]. Through an integrative taxonomic framework, we clarify the status of populations formerly identified as *O. minutus* across Fujian and Guangxi Zhuang Autonomous Regions, resulting in two concurrent taxonomic acts: (i) the synonymization of *O. minutus* with *O. bidens*, and (ii) the description of *O. baguiensis* sp. nov. as a species distinct from *O. bidens*.

### 4.1. Taxonomic Status of O. minutus

*Opsariichthys minutus* Nichols, 1926 [16] was originally described from Fujian. Subsequent to Chen [4], the taxon was widely regarded as a junior synonym of the more widespread *O. bidens*. Despite Huynh and Chen’s [8] attempting to revalidate the species, our comprehensive morphological comparison demonstrates extensive overlap between Fujian populations of ‘*O. minutus*’ and *O. bidens* across all diagnostic characters (Table 2 and Table 3). Minor variations, such as lateral-line scale counts (with minor differences) and predorsal scale counts (nearly identical), fall well within the range of intraspecific variation.

Molecular data strongly support this morphological conspecificity. Phylogenetic analyses of both D-loop and Cyt *b* sequences reveal that populations from the Minjiang, Jinjiang, Jiulongjiang, and Hanjiang river systems in Fujian cluster with *O. bidens* from the Pearl, Yangtze, Qiantangjiang, and adjacent river systems, forming a single, well-supported monophyletic lineage (Figure S1 and Figure 2) with minimal genetic divergence (uncorrected p-distance < 2%). Accordingly, we reaffirm the status of *O. minutus* as a junior synonym of *O. bidens*, based on the integrated molecular and morphological evidence presented above.

Synonymization does not imply that *O. minutus* is morphologically indistinguishable or difficult to recognize; rather, it reflects our conclusion that the Fujian populations represent the same biological and evolutionary entity as *O. bidens*, diagnosed here by extensive character overlap, zero fixed nucleotide differences, and minimal genetic divergence (<2% p-distance) (Figure 2 and Figures S1–S3). A limitation should be noted: no sequences from the type locality of *O. bidens* (Shanghai) were available, as the species is no longer extant there; our assessment therefore relied on geographically proximate populations (Jiangxi, Anhui, Zhejiang). This may leave a small margin of uncertainty regarding the conspecificity of Fujian populations with *O. bidens*, but the minimal genetic divergence and extensive morphological overlap strongly support the synonymization.

### 4.2. Recognition of O. baguiensis sp. nov.

In contrast, populations from the Xijiang River basin (middle and upper Pearl River system) in Guangxi Zhuang Autonomous Region—previously referred to as ‘*O. minutus*’ by Lin et al. [18] and Wang et al. [9]—constitute a distinct evolutionary lineage. Our results demonstrate that these Guangxi populations are both morphologically and genetically divergent from their congeners.

The species status is further substantiated by substantial genetic divergence. Uncorrected p-distances for Cyt *b* range from 7.4% to 15.9% between the new species and its congeners (Table S2). Notably, genetic divergence from sympatric *O. bidens* reaches 13.3%, while divergence from *O. hainanensis* is 7.4%, with phylogenetic reconstructions consistently recovering the new species as a well-supported sister group to *O. hainanensis* (Figure 2). Coupled with the pronounced geographic disjunction between the type locality of *O. minutus* (Fujian) and the Hongshuihe River basin (Guangxi), our findings indicate that the latter harbors a previously unrecognized lineage. The two regions are separated by a straight-line distance of approximately 900–1200 km. More critically, the Wuyi–Nanling mountain chain forms a biogeographic barrier between the Fujian drainages (where the *O. minutus* populations assigned to *O. bidens* occur) and the upper Xijiang basin in Guangxi, where the new species is endemic. Although *O. bidens* itself also occurs in the Xijiang basin, the Fujian populations and the Guangxi new-species lineage are disjunct across this barrier, supporting their recognition as distinct evolutionary lineages.

Morphologically, *Opsariichthys baguiensis* sp. nov. is distinguished by a prominent anterior protrusion on the lower jaw that articulates with a median notch on the upper jaw, producing undulating jaw margins (vs. straight jaw margins in Clade I/III congeners, Figure 2). While most similar to *O. bidens* and *O. hainanensis*, the new species differs consistently in several key characters: lateral-line scale count (49–55 vs. 44–47 in *O. bidens* and 41–43 in *O. hainanensis*); number of pharyngeal tooth rows (2 vs. 3); breeding tubercle arrangement (1–2 rows of prominent tubercles on lower jaw vs. 3–4 rows); relative snout length (36.5–40.1% HL vs. <32.1% HL in *O. bidens*); and caudal peduncle length (13.6–17.1% SL vs. >17.4% SL in *O. bidens*) (Table 1).

### 4.3. Biogeographic and Conservation Implications

The recovery of Clade III (comprising undulating-jawed *O. baguiensis* sp. nov., *O. hainanensis*, *O. dienbienensis*, *O. songmaensis*, plus multiple straight-jawed described congeners and reported lineages) as the sister group to Clade II (the *O. bidens* clade) suggests a complex biogeographic history within the Pearl River basin. The distinctiveness of *O. baguiensis* sp. nov., coupled with the co-occurrence of *O. bidens* and *O. rubriventris* (in Clade I; Xizhijiang River, Guangdong) in the Pearl River basin, suggests that the middle and upper Pearl River basin (specifically the Xijiang River system in Guangxi) may have supported localized *Opsariichthys* diversification, likely driven by historical river capture events or vicariance within the complex topography of Guangxi Zhuang Autonomous Region, a region whose drainage networks have been repeatedly reshaped by such processes since the late Cenozoic [29,30,31].

From a conservation perspective, *O. baguiensis* sp. nov. is currently known only from the Xijiang River basin, a system under substantial anthropogenic pressure from hydropower development and increasing shipping traffic. Given its narrow endemic distribution, the species may be vulnerable to habitat fragmentation and altered flow regimes, although no quantitative population assessment is yet available. We therefore recommend that its conservation status be evaluated (e.g., following IUCN Red List criteria) and that the Hongshuihe River type locality receive particular attention in future freshwater biodiversity monitoring. More broadly, the discovery of a previously unrecognized lineage underscores that the Pearl River basin’s fish diversity remains incompletely documented, and that ongoing development should be accompanied by systematic surveys to detect cryptic taxa before they are lost.

## 5. Conclusions

This study underscores the crucial role of integrative taxonomy in uncovering hidden diversity within *Opsariichthys*. By synonymizing *O. minutus* with *O. bidens* and describing *O. baguiensis* sp. nov., we refine the known distribution of the genus and highlight the middle and upper Pearl River basin (specifically the Xijiang River system) as a region of localized *Opsariichthys* diversification. Targeted future surveys across the Xijiang River system in Guangxi (including but not limited to the Hongshuihe River, Guijiang River and Liujiang River basins) are warranted to fully assess the distribution range and conservation status of this newly described species.

## 6. Comparative Material

*Opsariichthys bidens* (formerly identified as *O. minutus*): SHOU 20240624002, 1 specimen, 102.2 mm SL, Fuzhou City, Minjiang River System, Fujian Province, China; SHOU 20250106001, SHOU 20250106006–008, 4 specimens, 94.9–129.1 mm SL, Nanping City, Minjiang River System, Fujian Province, China; SHOU 20250110001, SHOU 20250110005, 2 specimens, 91.2–91.3 mm SL, Zhangzhou City, Jiulongjiang River System, Fujian Province, China.

*Opsariichthys hainanensis*: SHOU 20260206001–006, 6 specimens, 73.5–99.16 mm SL, Dongfang City, Changhuajiang River System, Hainan Province, China.

## 7. Nomenclatural Acts Registration

This published work and the nomenclatural acts it contains have been registered in ZooBank LSIDs (Life Science Identifiers). Publication LSID: urn:lsid:zoobank.org:pub:5E076276-5CBC-4015-86AB-5E107463EC9D. *Opsariichthys baguiensis* sp. nov. LSID: urn:lsid:zoobank.org:act:A74E068B-FE71-4AB8-9F25-EA663BA14980.

## Figures and Tables

**Table 1:** Key morphological differences between *Opsariichthys baguiensis* sp. nov. and four other congeners with undulating jaw margins.

Characters	*O. baguiensis* sp. nov	*O. bidens* *	*O. hainanensis*	*O. dienbienensis* *	*O. songmaensis* *
Lateral line scales	49–55	45–47	40–43	40–42	41–43
Scales above the lateral line	9–10	10	8	8–9	8–9
Predorsal scales	17–21	19–21	15–16	15–18	15–18
Pharyngeal teeth	2 rows	3 rows	3 rows	--	--
Snout length/head length (%)	36.5~40.1	29.3~32.1	28.0~30.1	29.2~30.7	32.5
Caudal peduncle length/standard length (%)	13.6~17.1	17.8~21.6	17.4~18.6	16.4~18.3	17.6
Lower jaw tubercles (mature males)	1–2 rows, densely packed, prominent	3–4 rows, well-separated, distinctively smaller	3 rows, well-separated, distinctively smaller	3–4 rows, well-separated, distinctively smaller	3–4 rows, well-separated, distinctively smaller
Cheek tubercles (mature males)	1–2 rows, densely packed, prominent	3–4 rows, well-separated, distinctively smaller	3 rows, well-separated, distinctively smaller	3 rows, well-separated, distinctively smaller	3 rows, well-separated, distinctively smaller

* data cited from literature: *O. bidens*, [10]; *O. dienbienensis*, [8]; *O. songmaensis*, [8].

**Table 2:** Frequency distribution of meristic characters of *Opsariichthys baguiensis* sp. nov. and five other congeners with undulating jaw margins, including ‘*O. minutus*’ (provisionally treated as distinct).

Species	Diii					P1i						P2i					
	7	8	M			13	14	15	M			7	8	9	M		
*O. baguiensis* sp. nov	28	-	7.0			13	15	-	13.5			-	19	9	8.3		
*O. bidens* *	10	-	7.0			-	10	-	14.0			-	10	-	8.0		
‘*O. minutus*’	7	-	7.0			-	7	-	14.0			-	7	-	8.0		
*O. hainanensis*	6	-	7.0			5	1	-	13.2			-	6	-	8.0		
*O. dienbienensis* *	10	-	7.0			3	6	1	13.8			2	8	-	7.8		
*O. songmaensis* *	10	-	7.0			7	3	-	13.3			4	6	-	7.6		
	**Aiii**					**LLa**						**LLb**					
	**9**	**10**	**M**			**8**	**9**	**10**	**M**			**3**	**4**	**5**	**M**		
*O. baguiensis* sp.nov	25	3	9.1			-	22	6	9.2			-	28	-	4.0		
*O. bidens* *	1	9	9.9			-	-	10	10.0			3	6	1	3.8		
‘*O. minutus*’	-	7	9.0			-	-	7	10.0			1	4	2	4.2		
*O. hainanensis*	6	-	9.0			6	-	-	8.0			-	6	-	4.0		
*O. dienbienensis* *	10	-	9.0			2	8	-	8.8			-	10	-	4.0		
*O. songmaensis* *	9	1	9.1			2	8	-	8.8			-	10	-	4.0		
	**CPS**									**PreD**							
	**15**	**16**	**17**	**18**	**19**	**20**	**M**			**15**	**16**	**17**	**18**	**19**	**20**	**21**	**M**
*O. baguiensis* sp. nov	1	6	11	7	1	3	17.9			-	-	3	8	10	5	2	18.8
*O. bidens* *	-	-	2	7	1	-	17.9			-	-	-	-	3	5	2	19.9
‘*O. minutus*’	-	-	5	1	1	-	17.4			-	-	-	-	2	4	1	19.9
*O. hainanensis*	-	1	3	2	-	-	17.2			-	3	3	-	-	-	-	16.5
*O. dienbienensis* *	-	1	4	3	2	-	17.6			1	4	3	2	-	-	-	16.6
*O. songmaensis* *	-	-	2	5	3	-	18.1			6	2	1	1	-	-	-	15.7
	**LL**																
	**40**	**41**	**42**	**43**	**44**	**45**	**46**	**47**	**48**	**49**	**50**	**51**	**52**	**53**	**54**	**55**	**M**
*O. baguiensis* sp. nov	-	-	-	-	-	-	-	-	-	4	5	3	9	2	2	3	51.6
*O. bidens* *	-	-	-	-	-	2	6	2	-	-	-	-	-	-	-	-	46.0
‘*O. minutus*’	-	-	-	-	-	2	4	1	-	-	-	-	-	-	-	-	45.9
*O. hainanensis*	3	2	1	-	-	-	-	-	-	-	-	-	-	-	-	-	40.7
*O. dienbienensis* *	2	3	5	-	-	-	-	-	-	-	-	-	-	-	-	-	41.3
*O. songmaensis* *	-	2	7	1	-	-	-	-	-	-	-	-	-	-	-	-	41.9

* data cited from literature: *O. bidens*, [10]; *O. dienbienensis*, [8]; *O. songmaensis*, [8].

**Table 3:** Morphometric measurements of *Opsariichthys baguiensis* sp. nov. and three other congeners with undulating jaw margins with available data.

	*O. baguiensis* sp. nov.							*O. bidens* *						‘*O. minutus*’ *= O. bidens*						*O. hainanensis*					
	Holotype Male				Female			Male			Female			Male			Female			Male			Female		
N	1	19			8			2			8			4			3			5			1		
Standard length (mm)	107.4	95.2~120.6			85.6~102.4			95.2~100.6			76.1~131.6			102.2~129.1			91.2~94.9			73.5~84.4			99.16		
% in SL		Min	Max	Mean	Min	Max	Mean	Min	Max	Mean	Min	Max	Mean	Min	Max	Mean	Min	Max	Mean	Min	Max	Mean	Min	Max	Mean
Body depth	20.7	18.4	25.9	22.7	20.4	23.7	22.1	24.6	26.8	25.7	20.1	25.2	22.5	23.0	31.3	26.4	20.2	24.7	23.1	22.5	24.4	23.9	21.3	21.3	21.3
Head length	26.8	26.7	30.1	28.2	27.7	30.0	28.6	30.0	30.1	30.0	29.8	31.1	30.5	28.7	32.2	30.6	29.5	30.7	29.9	28	31.5	29.5	27	27	27
Caudal peduncle length	16.2	13.6	17.1	15.7	14.7	16.7	15.8	17.3	18.2	17.7	14.6	20.0	16.5	17.1	19.4	18.0	16.7	17.4	17.0	17.4	18.6	18.0	17.5	17.5	17.5
Caudal peduncle depth	9.1	7.6	9.9	8.8	8.0	9.5	8.6	9.0	9.5	9.2	8.2	9.9	8.8	8.9	11.1	9.8	8.9	9.4	9.1	9.2	10.6	10.1	8.9	8.9	8.9
Dorsal fin length	21.0	15.9	23.4	19.8	17.0	19.5	17.8	18.7	18.9	18.8	14.1	17.4	15.8	21.0	24.8	23.2	18.1	20.6	19.7	24.8	28.0	25.6	19.9	19.9	19.9
Pectoral fin length	20.1	14.0	21.9	20.3	16.8	19.7	18.4	19.6	22.0	20.8	12.9	19.8	17.9	20.0	22.9	21.7	17.6	18.7	18.2	21.9	23.3	22.3	18.9	18.9	18.9
Pelvic fin length	14.8	13.7	17.1	15.6	12.8	15.3	13.6	15.3	16.2	15.7	11.7	14.8	13.6	15.9	17.6	16.9	13.9	14.2	14.0	14.7	17.2	15.9	14	14	14
Anal fin length	31.0	25.9	34.3	31.0	20.1	25.5	23.5	25.6	28.5	27.0	18.3	25.1	21.8	32.0	36.2	34.6	24.5	29.8	27.4	29.8	34.1	32.2	25.7	25.7	25.7
Dorsal fin base length	11.3	10.2	12.7	11.2	10.0	10.6	10.3	13.0	13.3	13.2	9.3	11.4	10.6	11.7	12.8	12.3	11.1	11.8	11.5	13.5	15.1	14.3	11.2	11.2	11.2
Pectoral fin base length	4.6	4.1	6.2	5.0	4.3	5.6	4.8	4.5	5.1	4.8	3.3	4.4	3.9	4.6	5.8	5.2	3.9	4.4	4.0	5.4	6.7	5.9	4.9	4.9	4.9
Pelvic fin base length	4.1	3.5	5.3	4.2	4.0	4.4	4.2	3.7	3.8	3.7	3.2	4.0	3.6	3.9	4.4	4.2	3.6	4.4	4.0	4.0	5.7	4.7	4.0	4.0	4.0
Anal fin base length	14.6	12.0	16.0	14.5	12.6	13.0	12.9	15.1	15.4	15.3	10.5	12.0	11.0	13.0	15.7	14.5	12.2	13.7	13.0	15.9	17.6	16.7	13.5	13.5	13.5
Predorsal length	52.4	51.4	55.6	53.5	53.5	56.3	54.6	53.2	53.3	53.2	51.8	56.2	53.8	50.0	56.0	53.4	54.0	55.9	55.1	49.8	52.7	51	49.1	49.1	49.1
Prepectoral length	25.2	25.3	30.0	27.3	27.5	29.0	28.3	26.8	27.0	26.9	27.5	29.6	28.9	26.7	29.3	27.9	26.8	27.8	27.3	24.8	27.5	26.3	25.1	25.1	25.1
Prepelvic length	50.5	50.1	53.5	51.9	50.5	53.3	52.2	50.1	52.0	51.1	51.6	54.8	53.0	51.4	55.3	53.3	52.1	53.0	52.5	51.1	54.3	52.6	49.5	49.5	49.5
Preanal length	72.9	69.3	73.0	71.2	71.2	76.1	72.7	68.9	69.3	69.1	71.2	73.9	72.6	70.3	71.9	70.9	72.0	73.7	72.7	69.4	71.7	70.9	70.2	70.2	70.2
% in HL																									
Head depth	66.1	59.6	70.2	65.3	62.1	64.0	63.0	64.0	66.2	65.1	56.4	62.6	59.4	65.9	69.9	68.3	54.0	65.0	59.4	66.4	75.2	69.6	70	70	70
Head width	48.1	43.7	51.1	46.9	43.1	45.4	44.3	49.0	54.3	51.6	38.9	49.5	43.9	47.9	50.2	49.1	41.4	48.3	45.6	45.4	51.2	47.7	48.6	48.6	48.6
Snout length	37.4	36.5	40.1	38.1	33.9	38.4	36.1	29.3	32.1	30.7	29.6	33.7	32.1	31.7	35.9	33.9	31.7	35.0	33.8	28	30.1	29.4	30.9	30.9	30.9
Eye diameter	20.1	17.2	23.1	20.4	20.6	23.5	22.2	18.2	19.6	18.9	16.0	22.4	19.1	15.7	18.0	16.4	18.8	19.8	19.3	20.3	26.7	23.9	22.7	22.7	22.7
Interorbital width	34.3	30.2	36.4	33.1	30.8	33.1	32.3	31.7	31.9	31.8	28.2	31.4	29.7	31.3	32.5	32.1	30.6	31.3	30.8	28.7	35	31.5	31.7	31.7	31.7

* data cited from literature: *O. bidens*, [10].

## Data Availability

The original genetic data presented in the study are openly available in NCBI. All other data are available within the Supplementary Materials of the paper.

## Supplementary Materials

The following supporting information can be downloaded at: https://www.mdpi.com/article/10.3390/ani16203193/s1, Figure S1. Phylogenetic relationships of *Opsariichthys* specimens with undulating jaw margins inferred from mitochondrial D-loop sequences. The ML tree is shown; BI analysis recovered congruent topologies. Nodal support values represent ML bootstrap proportions/BI posterior probabilities. The *O. bidens* assemblage (including Fujian populations) and the new species are shaded light green and tan, respectively; drainage-specific colors are given in the legend at right. Asterisks (*) denote the two Fujian *O. minutus* D-loop sequences of Huynh and Chen [8] retrieved from GenBank, used to anchor specimens to the *O. minutus* lineage. Figure S2. Two-way hierarchical clustering heatmap of 18 shared morphological traits across six *Opsariichthys* species (Z-score standardized; Euclidean distance, complete linkage). *O. baguiensis* sp. nov. forms a distinct cluster separated from the other five species, supporting the treatment of ‘*O. minutus*’ as a junior synonym of *O. bidens*. Figure S3. PCA score plot of four *Opsariichthys* species based on 21 morphometric variables. *O. baguiensis* sp. nov. is separated from the other groups; ‘*O. minutus*’ and *O. bidens* overlap and are not distinguishable. Table S1. Specimen data and collection localities for samples used in phylogenetic analyses in this study. References [32,33] are cited in Table S1 for the sequence sources. Table S2. Mean interspecific Cyt *b* genetic distances (uncorrected p-distance) among *Opsariichthys*.
