# Supplementary material for: Integrative Taxonomy Reveals a New Species of Opsariichthys (Teleostei, Cypriniformes) from the Pearl River Basin in Guangxi Zhuang Autonomous Region, China, with Synonymization of O. minutus
Source: Animals (Basel). 2026 Oct 11;16(20):3193. doi: 10.3390/ani16203193 (PMC13700678; doi:10.3390/ani16203193)
Supplement: Supplementary file 1 [file animals-16-03193-s001.zip › Supplementary Figures S1-3.pdf]

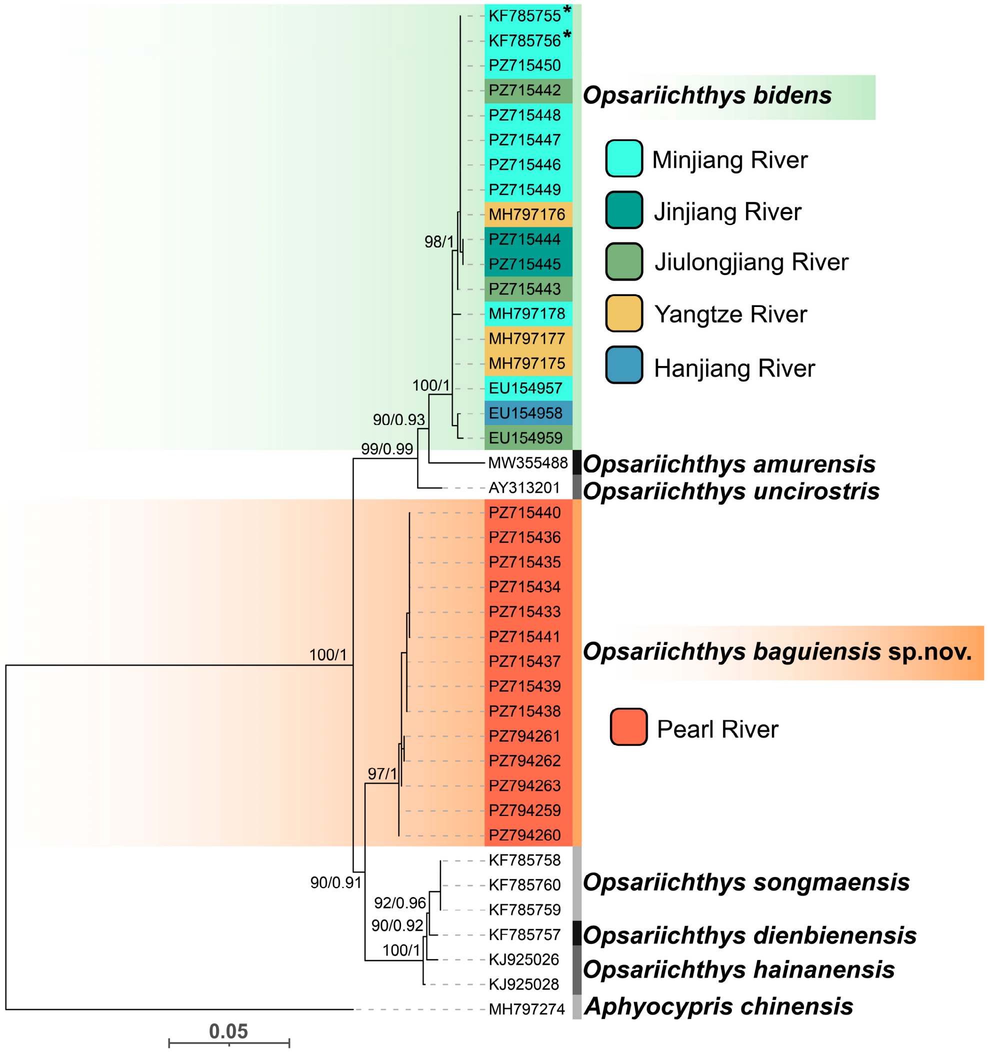

Figure S1. Phylogenetic relationships of *Opsariichthys* specimens with undulating jaw margins inferred from mitochondrial D-loop sequences. The ML tree is shown; BI analysis recovered congruent topologies. Nodal support values represent ML bootstrap proportions/BI posterior probabilities. The O. *bidens* assemblage (including Fujian populations) and the new species are shaded light green and tan, respectively; drainage-specific colors are given in the legend at right. Asterisks (*) denote the two Fujian *O. minutus* D-loop sequences of Huynh and Chen (2013) [8] retrieved from GenBank, used to anchor specimens to the *O. minutus* lineage.

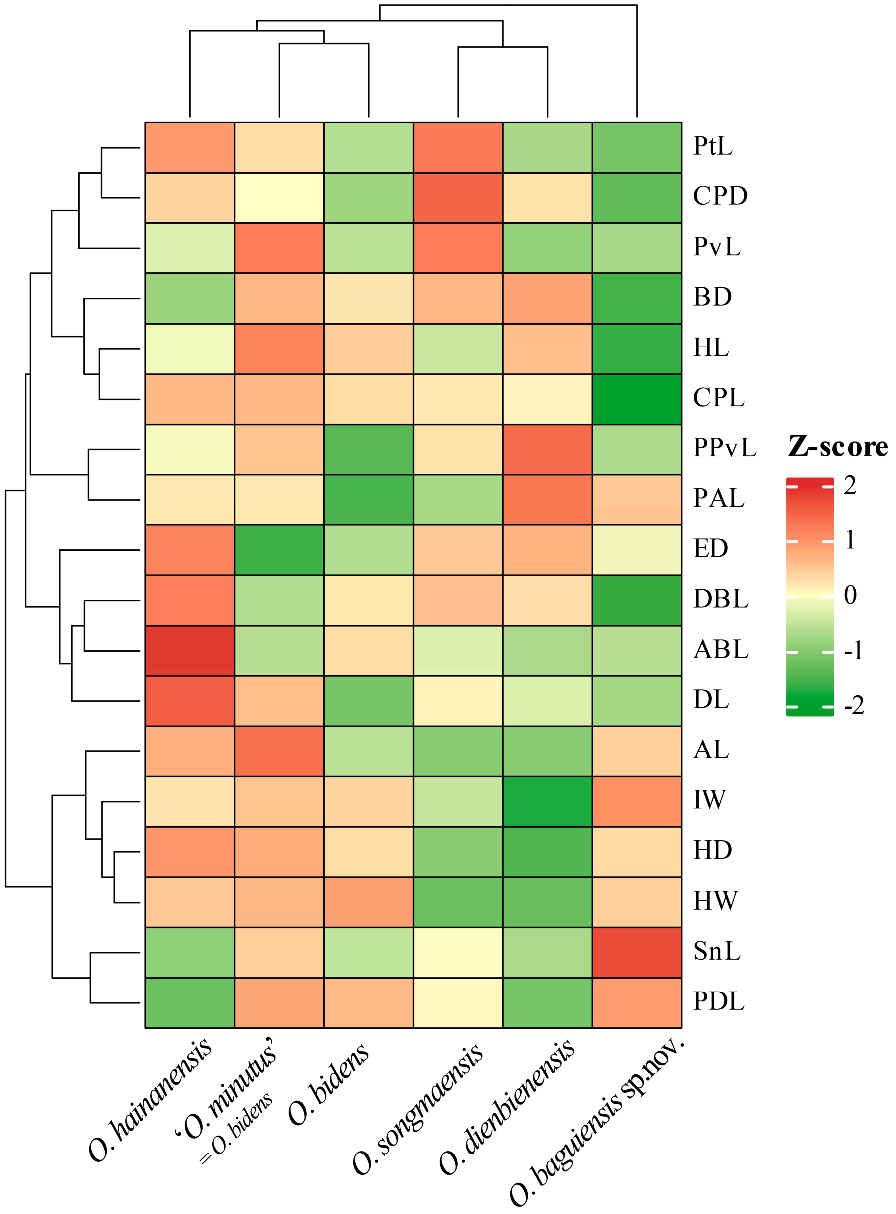

Figure S2. Two-way hierarchical clustering heatmap of 18 shared morphological traits across six *Opsariichthys* species (Z-score standardized; Euclidean distance, complete linkage). *O. baguiensis* sp. nov. forms a distinct cluster separated from the other five species, supporting the treatment of '*O. minutus*' as a junior synonym of *O. bidens*.

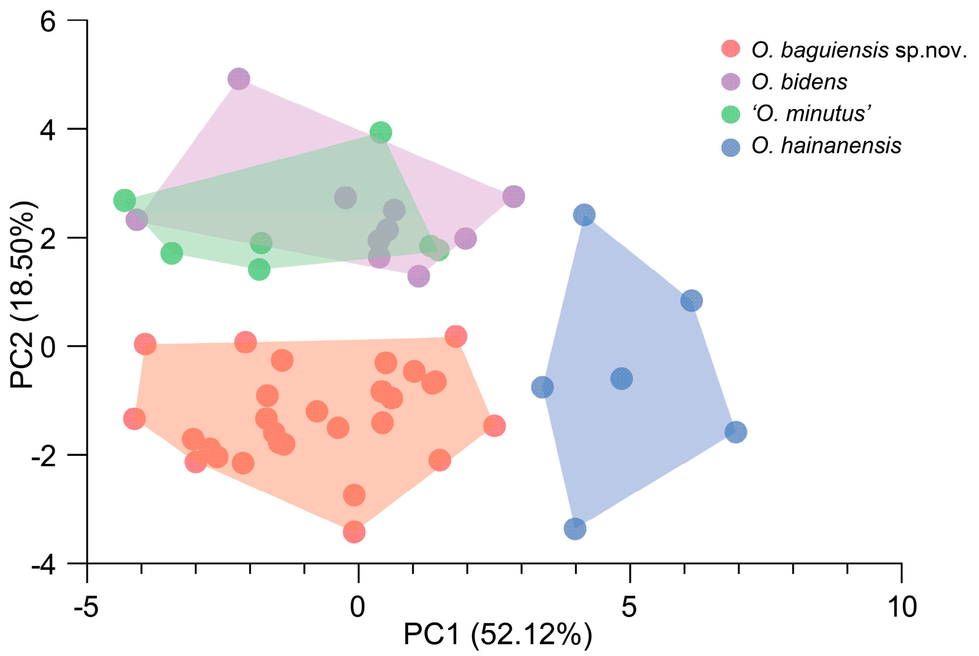

Figure S3. PCA score plot of four *Opsariichthys* species based on 21 morphometric variables. *O. baguiensis* sp. nov. is separated from the other groups; '*O. minutus*' and *O. bidens* overlap and are not distinguishable.
